# Male involvement in prevention of mother to child transmission of human immunodeficiency virus and associated factors in Enebsiesarmider District, north West Ethiopia, 2018: a cross-sectional study

**DOI:** 10.1186/s12884-020-2837-y

**Published:** 2020-03-06

**Authors:** Haimanot Abebe Adane, Nega Assefa, Bizatu Mengistie, Asmamaw Demis

**Affiliations:** 1 Department of Public Health, College of Health Sciences and Medicine, Wolkite University, Wolkite, Ethiopia; 2 Department of Public Health, College of Health Sciences and Medicine, Haramaya University, Harar, Ethiopia; 3 Department of Nursing, College of Health Sciences, Woldia University, Woldia, Ethiopia

**Keywords:** Male involvement, Northwest Ethiopia, Prevention of mother-to-child transmission of HIV

## Abstract

**Background:**

Globally, male involvement has been identified as a priority target area to be strengthened in the prevention of mother to child transmission (PMTCT) of HIV. However, there are limited studies on husband involvement in the prevention of mother to child transmission of HIV in Ethiopia. Therefore, this study aimed to assess male involvement in the prevention of mother to child transmission of HIV and associated factors among males whose wives gave birth in the last six months before the survey in Enebsiesarmider district, Northwest Ethiopia.

**Methods:**

A Community-based cross-sectional study was employed to assess male involvement in the prevention of mother to child transmission of human immunodeficiency virus and associated factors in Enebsiesarmider District, Northwest Ethiopia. The study was conducted from February 10–30, 2018. A total of 525 participants were involved in the study. A stratified cluster sampling method was used to recruit study participants. Data were collected using a structured interviewer-administered questionnaire. Data were entered using the epi Data software and exported to SPPS for analysis. Descriptive statistics including mean, a proportion were used to describe study variables. Multivariable logistic regression was employed to describe variables with the outcome variable.

**Result:**

Overall male involvement in PMTCT was found to be 26.1% [95%CI, 22.1–29.5]. Respondents who have attended secondary education and above were more likely get involved in PMTCT than who have no formal education [AOR 2.45, 95%CI, 1.47–4.11], Respondents who have good knowledge on PMTCT [AOR 2.57, 95%CI, 1.58–4.18], good knowledge on ANC [AOR 2.10, 95%CI, 1.28–3.44], low cultural barriers [AOR 2.20, 95%CI, 1.34–3.63] low health system barriers [AOR 2.40, 95%CI, 1.37–4.20] were variables that significantly increase male involvement in PMTCT practices.

**Conclusion:**

Male involvement in PMTCT was found to be low in the study area. Therefore, the district health office in collaboration with local health care providers shall design strategies for enhancing male involvement through creating a husband’s knowledge regarding the merit of prevention of mother to child transmission through the provision of adequate information for all male partners at ANC clinic is recommended.

## Background

Human Immune Deficiency Virus (HIV) can be transmitted from an HIV infected mother to her child during pregnancy, labor or delivery, and breastfeeding. The aforementioned mode of transmissions is collectively known as mother-to-child transmission of HIV or vertical transmission of HIV [1–3]. Preventing HIV transmission in pregnant women and their children often referred to as the prevention of mother-to-child transmission [4, 5]. Mother-to-child transmission of HIV remains a significant problem in the low and middle-income countries, despite the development and growing availability of effective prevention methods for resource-limited settings [6, 7].

Male involvement in sexual and reproductive health has recently been recognized as new strategy for enhancing maternal and child health by playing a role in preventing women’s risk of acquiring HIV, but also in terms of her utilization of the PMTCT program: for the mother to test for HIV, to return for the result, for the couple to use condoms, to receive medication, and to increase adherence to proper infant feeding practices [7, 8]. Ethiopia has adopted the global target for PMTCT which is nullifying mother to child transmission by the year 2030 [9]. For the aforementioned ambitious goal, male involvement is highly demanded especially in low and middle-income countries where the community is patriarchal.

However, male involvement in the PMTCT program remains low in low and middle-income countries, including Ethiopia [10, 11]. For instance, a study conducted in the Myanmar region of Asia revealed that male involvement in PMTC is 13% [12], A Brief Review of Initiatives in East, West and Central Africa showed that male involvement in PMTCT was low with a range from 1.8 to 32% [13]**.** Another systemic review conducted in Ethiopia showed that male involvement in PMTCT was 14 to 30% [11]. Recent studies on the topic suggest that there were several reasons for the low level of male involvement, including cultural **barriers** and norms, PMTCT/ANC knowledge, sociodemographic characteristics, male individual factors and health system [13–15].

This low level of male involvement in the prevention of mother to child transmission of HIV results in pregnant women not to get tested for HIV [16]. Those who dared to go for a test, if tested positive were afraid to disclose their serostatus to their husbands because they thought their husbands would accuse them of infidelity, face divorce and violence, some were not even allowed to continue with PMTCT interventions and increase in maternal to child transmission of HIV -[17].

Ethiopia has made major efforts through the scaleup of the PMTCT program, including reinforcing messages in well-designed community mass media campaigns, create an opportunity for constructive dialogue between men and women, forming women and male health development army and making services more male-friendly [18]. Few studies have been conducted from the male point of view for their low participation in PMTCT in Ethiopia [19–21]. In the study area, there is no study conducted so far on male involvement in PMTCT that could be attributable to the male involved in the uptake of PMTCT service. Therefore, this study would address this gap by assessing the involvement of male partners in PMTCT and its barriers at a community level in Enebsiesarmider District.

## Materials

### Study area and study period

The study was conducted in Enebsiesarmider district Northwest Ethiopia from February 10–30/2018. The administrative center of this district is Mertolemariam, which is located 364 Km to the North of Addis Ababa, the capital city of Ethiopia. Based on the 2007 census population projection, the total population of the district was 162,132, of which (male accounts 78,481 and female accounts 83,651. Regarding the health service, the district has 1 primary hospital, 8 health centers, 38 health posts, 4 drug vendors and 5 private clinics. The annual report from Enebsiesarmider District office in 2017 indicated that the health coverage of institutional delivery was 62%, ANC coverage 67.6% and PMTCT uptake 69%.

### Study design and study population

The community-based cross-sectional study design was conducted to assess male involvement in PMTCT and associated factors among males whose wives have given birth in the last six months before the survey. All males whose wives had ANC follow up during their last pregnancy and have lived in the study area for at least six months were eligible for the study.

### Sample size determination and sampling procedures

The required sample size for this study was calculated by using single population proportion formula: With assumption of 95% of confidence interval**,** margin of error tolerated (0.05)**,** the proportion of male involvement in PMTCT 0.309 [19], non-response 10% and design effect 1.5 to accommodate for intracluster variability, the maximum sample size was 542.

A stratified cluster sampling technique was employed to recruit study participants. The strata are urban and rural administrative units of the study area while the cluster is kebele (the smallest administrative unit in Ethiopia). The district has 37 kebeles (33 rural and 4 urban). The first sample size was proportionally allocated to urban and rural kebeles which results in 61 urban and 481 rural male allocations. Then the required numbers of kebeles that can accommodate the allocated sample to each stratum were selected by a simple random sampling technique. These results in 1 urban and 4 rural kebeles to be involved as actual data collection sites.

After that, the number of males whose wives gave birth in the last six months found was taken from a family folder which was documented by the health extension workers (HEWs) with their household. Finally, all males whose wives gave birth in the last six months in selected Kebles were studied.

### Operational definitions

#### Male involvement

##### Male involvement

Was measured using ‘Yes’, ‘No’ questions, Respondents were asked “did you know your wife’s ANC appointment the last time she was pregnant”; respondents who respond “yes” will score 1 and “No” response will earn zero scores. The same pattern of questioning and scoring was made for the rest of the five PMTCT male involvement questions. According to the aforementioned statements, the involvement score for each respondent could range from 0 to 6. A total score of 4–6 was considered as “involved in PMTCT” and scores of 0–3 are labelled as not involved in PMTCT [22].

##### Cultural **barriers**

Was measured using five-point Likert scale questions, Respondents were asked “did you believe PMTCT information should first be given to men than to women”; respondents who respond “very disagree” will score 1, “disagree” will score 2, “undecided” will score 3, agree will score 4 and “very agree” response will earn 5 score. The same pattern of questioning and scoring was made for the rest of the nine cultural **barriers** items. According to the aforementioned statements, the score for each respondent could range from 10 to 50. Finally, respondents who have scored greater than or equal to the mean value of the items have ‘High cultural **barriers**’ and those who have scored less than the mean value of the items have “Low cultural **barriers**’ [23].

##### Health system **barriers**

Was measured using five-point Likert scale questions, Respondents were asked “did you believe antenatal clinics should be opened on weekends and evening for men to attend these clinics with their partner”; respondents who respond “very disagree” will score 1, “disagree” will score 2, “undecided” will score 3, agree will score 4 and “very agree” response will earn 5 score. The same pattern of questioning and scoring was made for the rest of the seven health system **barriers** items. According to the aforementioned statements, the score for each respondent could range from 8 to 40. Respondents who have scored greater than or equal to the mean value of the items have ‘High health system **barriers**’ and those who have scored less than the mean value of the items have “Low health system **barriers’** [23].

##### PMTCT knowledge

Comprehensive knowledge of PMTCT was computed by summing up all relevant 10 knowledge items (item on ever heard about PMTCT Service, about MTCT of HIV transmission and prevention method). A correct answer for each item was scored as “1” and an incorrect answer was scored as “0.” Items were then summed up and converted into 100%. Accordingly, those respondents who scored greater than 60% of knowledge assessment questions were thought of as having Good knowledge and those respondents who answered less than 60% of knowledge assessment questions were thought of as having Good knowledge [24].

##### Socio-demographic factors

The demographic factors are age, education level, occupation, religion, residence, and income.

### Data collection tool and procedure

The data collection tool was developed by reviewing different work of literature [19–21] and it consists of five parts sociodemographic characteristics, knowledge related factors, cultural and programmatic **barrier** related factors, male individual-related factors and level of male involvement in PMTCT. The English language questionnaire was translated into Amharic language (language spoken in the study area) by an Amharic language speaker who has attended the Master of Arts in Amharic language and was translated back to English language by a person who attended Master of Arts in English language and comparison was made on the consistency of the two versions. The questions are both open and close-ended. Cultural and programmatic barrier assessment tools were adopted from a study done in Uganda and central Ethiopia [22, 23]. Cultural barriers were assessed by ten reliable items (Cronbach’s alpha = 0.903) and a programmatic barrier was assessed by eight reliable items (Cronbach’s alpha = 0.810) to ensure the reliability of the scale [24].

The data were collected by 10 grade 10 completed students who are fluent in the local language (Amharic) and familiar with the study area. The data collection process was supervised by three BSc holders (Midwives). Data were collected via house to house visit by trained data collectors; For respondents who are not available at home at the first visit, a revisit was arranged at a minimum of three times.

### Data quality control

To assure the quality of the data, a structured and pretested questionnaire was used. Two days of training were given for data collectors and supervisors about techniques of data collection, instruments, ethical issues and purpose of the study. Intensive supervision was done by the principal investigator and supervisor. The collected data were checked for completeness, accuracy, and consistency throughout the data collection period.

### Data processing and analysis

The data were coded, cleaned, edited and entered into Epi data version 4.2 and exported to SPSS window version 24 for analysis. Descriptive results were presented using tables and figures. Model fitness was checked using a Hosmer–Lemeshow goodness-of-fit test. Crude odds ratios with their 95% confidence intervals were estimated in the bi-variable logistic regression analysis to assess the association between each independent variable and outcome variable. All variables with *P* ≤ 0.25 in the bivariate analysis were included in the final model of multivariate analysis to control all possible confounders. The adjusted odds ratio with 95% CI was estimated to identify the factors associated with male involvement in PMTCT using multivariable logistic regression analysis. The level of statistical significance was declared at *P*-value< 0.05.

## Result

### Socio-demographic characteristics of study participants

A total of 525 study participants were involved in this study, making a response rate of 96.9%. The mean age of study participants was 35.7 (SD ± 6.4 years). All, 525 (100%) of respondents were Orthodox followers by religion and Amhara by ethnicity. More than four-fifth, 466 (88.8%) of male partners were from rural areas. More than two fifths, 230 (43.8%) of the respondents were unable to read and write. The majority, 454(86.5%) of the study participants were farmers by their occupation. Regarding the family wealth index, nearly two-fifth 200 (38.1%) of households were 2nd quintile wealth range (See Table 1).

**Table 1:** Sociodemographic characteristics of study participants in Enebsiesarmider District, Northwest, Ethiopia, 2018(*n* = 525)

Variable	Frequency	Percent
Age (Year)		
≤ 25	14	2.7
26–35	266	50.6
≥ 36	245	46.7
Residence		
Urban	59	11.2
Rural	466	88.8
Education		
Unable to read and write	230	43.8
Able to read and write	109	20.1
Grade 1–8	64	12.8
Grade 9–12	96	18.3
College and above	26	5.0
Occupation		
Farmer	454	86.5
Merchant	31	5.9
Government Employee	21	4.0
Private Employee	8	1.5
Daily Laborer	11	2.1
Family wealth index		
1st Quintile	152	29.9
2nd Quintile	200	38.1
3rd quintile	173	32.0

### PMTCT and ANC knowledge of respondents

**Fig. 1:**
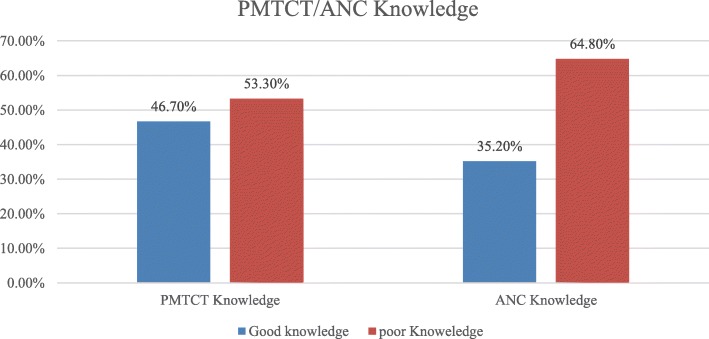
PMTCT and ANC knowledge among respondents, in Enebsiesarmider District, East Gojjam Zone, Ethiopia, 2018(n = 525)

Regarding PMTCT knowledge of participants, two hundred forty-five (46.7%) of them had good knowledge. Concerning ANC knowledge of respondents, it was calculated by summing up seven ANC questions and the mean value was calculated. Finally, those respondents scored mean and above were categorized as having good knowledge about ANC. Almost one third, 185(35.2%) of them had good ANC knowledge (Fig. 1).

### Cultural and health system variables of study participants

Regarding cultural **barriers (**factors relates to men’s opinion, perception, and the role that hinder the involvement of males in PMTCT), the mean sum scores of cultural **barriers** range from 10 to 46 with a median of 30 and mean of 27.15 with a standard deviation of 9.4. Three hundred two (57.5%) of participants had high cultural **barriers**. Concerning health system **barriers**, the mean sum scores of health system **barriers** range from 9 to 38, with a median of 21.0, mean of 22.03 and a standard deviation of 7.4. Based on this, 281(53.3%) of the respondents had low health system **barriers**.

### Male individual variables

Among respondents, 225 (42.9%) of them had a fear of disclosure of HIV results to their wives. Almost two fifths, 211 (40.2%) of the participants were bothered to know what goes on in ANC. Only fifty-three (10.1%) of the respondents had gone together for VCT with his wife. More than three-fifths of respondents, 329 (62.7%) knew their HIV serostatus.

### Level of male involvement in PMTCT

One hundred sixty-two (30.9%) of the 525 men had attended ANC with their partners. Nearly one fourth, 123(23.4%) of respondents were new their partners ANC appointment. Most of them, 471(89.7%) provided financial support to their spouses to attend ANC. Almost half of the respondents, 276 (52.6%) were willing to use condoms during sexual intercourse for PMTCT of HIV. Among the total respondents only 137 (26.1%) of them, had male involvement in PMTCT.

### Factor associated with male involvement in PMTCT services

Multivariable analysis showed that the odds of male involvement in PMTCT were 2.45 [AOR 2.45, 95%CI, 1.47–4.11] times higher in males who attended secondary and above education than those who had no formal education. Those males who had good knowledge about PMTCT and ANC program were 2.57 [AOR 2.57, 95%CI, 1.58–4.18] and 2.10 [AOR 2.10, 95%CI, 1.06–3.84] times more likely involved in PMTCT than those who had poor knowledge respectively. Regarding cultural **barriers**, those males who had low cultural **barriers** were 2.20 [AOR 2.20, 95%CI, 1.34–3.63] times more likely to be involved in PMTCT than those who had high cultural **barriers** and those respondents who had low health system **barriers** were 2.40 [AOR 2.40, 95%CI, 1.37–4.20] times more likely involved in PMTCT services when compared with those who had high health system **barriers (See** Table **2****)**.

**Table 2:** Factors associated with male involvement in PMTCT of HIV among male partners (crude and adjusted OR) in Enebsiesarmider District, Northwest Ethiopia, 2018 (n = 525)

Variables	Involved in PMTCT		COR (95%)	AOR (95%)
	Yes (%)	No (%)		
Educational status				
No formal education	60 (17.7)	279 (82.3)	1.00	1.00
Primary education	22 (34.4)	42 (65.6)	2.44 (1.36–4.38)	2.02 (1.06–3.84)*
Secondary education and above	55 (45.1)	67 (54.9)	3.82 (2.43–6.00)	2.45 (1.47–4.11)*
Residences				
Urban	24 (40.7)	35 (59.3)	2.14 (1.22–3.75)	1.75 (0.84–3.64)
Rural	113 (24.2)	353 (75.8)	1.00	1.00
Wife place of delivery				
Institution	89 (32.2)	187 (67.8)	1.99 (1.61–3.68)	1.48 (0.93–2.37)
Home	48 (19.3)	201 (80.7)	1.00	1.00
PMTCT knowledge				
Good	99 (40.4)	146 (59.6)	4.32 (2.82–6.62)	2.57 (1.58–4.18)**
Poor	38 (27.7)	242 (86.4)	1.00	1.00
ANC knowledge				
Good	76 (41.1)	109 (58.9)	3.19 (2.13–4.77)	2.10 (1.28–3.44)***
Poor	61 (17.9)	279 (82.1)	1.00	1.00
Go with a spouse for VCT				
Yes	18 (34.0)	35 (66.0)	1.53 (0.83–2.79)	1.96 (0.86–4.48)
No	119 (25.2)	353 (74.8)	1.00	1.00
Know HIV serostatus				
Yes	95 (28.9)	234 (71.1)	1.49 (0.98–2.26)	1.29 (0.86–4.50)
No	42 (21.4)	154 (78.6)	1.00	1.00
Cultural **Barriers**				
Low	89 (39.9)	134 (60.1)	3.51 (2.24–5.29)	2.20 (1.34–3.63)****
High	48 (15.9)	254 (84.1)	1.00	1.00
Health system **Barriers**				
Low	101 (35.9)	180 (64.1)	3.24 (2.11–4.98)	2.40 (1.37–4.20)*****
High	36 (14.8)	208 (85.2)	1.00	1.00

Significant at *P = 0.032, **P = 0.001, ***P = 0.000, ****P = 0.003, *****P = 0.002, ******P = 0.002, 1.00 = constant

## Discussion

The overall male involvement in PMTCT was found to be 26.1% [95%CI, 22.1–29.5]. This finding is quite low compared to what it should be. This finding implied that there is an unfinished agenda that district health office and local health care providers could work together to achieve full-scale male participation in PMTCT. It calls for urgent action to incorporate the important role of the male in preventing mother to child transmission of HIV in the study area specifically and in Ethiopia in general. This finding is in line with a study report from Eastern Uganda, Addis Ababa, Ethiopian and Tanzania [22, 23, 25]. This study was slightly higher than a study conducted in Gonder and Eastern Oromia Ethiopia [20, 26]. The difference could probably be explained by the difference in the time gap as better attention has been given to male involvement in PMTCT these days, improvements in the health care systems because the Ethiopian government gives great emphasis on maternal and newborn health which leads to higher involvement in PMTCT. The finding in this study indicated that there is already a crated platform for male participation in the study area, and this could serve as a springboard to achieve full-scale male participation in PMTCT in Enebsiesarmider district.

However, this finding is lower when compared with the study from Nepal, Thailand, and South Africa [26–29] and Hadiya (Lemo District), Arbaminch Ethiopia [19, 21]. The discrepancy of these findings might be attributed to the difference in methods used and study settings, sociodemographic characteristics of the study participants, and availability and accessibility of health service infrastructures. This finding implies that there is a gap that the district health office and regional health bureau could work in collaboration with the local health care provider to enhance the involvement of husbands in the prevention of mother to child transmission.

Males who had primary, secondary and above education were 2.02 and 2.45 times more likely to be involved in PMTCT than those who had no formal education respectively. Similar studies in Uganda, Nepal, Gonder, Arbaminch, and Debremarkos Ethiopia elsewhere have found that education level is an important factor of involvement in PMTCT services [20–22, 27, 30]. This may be related to as people more educated; they could easily understand both the transmission and prevention methods of HIV from mother to child. Moreover, educated males will have better awareness about the benefits of preventive health care including PMTCT and higher receptivity to new health-related information. Overall, as males receive more education, the chance may be higher because they could understand their role in sexual and reproductive health.

In this study male who had good knowledge about the PMTCT program were 2.57 times more likely involved in PMTCT than those who had poor PMTCT knowledge. This is consistent with the results of a study done in Arbaminch, Gonder Ethiopia and Tanzania, [20, 21, 25]. The possible explanation for this might be having good knowledge about PMTCT will help the partners to know the benefit of the PMTCT program, to involve in a discussion about safer sexual practices and assisting HIV positive pregnant women to get clinics. And as the male partners had good PMTCT knowledge about when does HIV transmission occurs from mother to baby, risk factors that increase the risk of HIV transmission during pregnancy and PMTCT core interventions the husband would have involved in PMTCT.

Males who had good knowledge of ANC services were 2.10 times more likely involved in the PMTCT program than those who had poor ANC knowledge. This finding is similar to a study conducted in Arbaminch, Ethiopia [21]. This could be since male partners who had good ANC knowledge will have information that could assist them in making decisions regarding healthy behaviors including sexual and reproductive health education and promotion.

Cultural **barriers** were also found to be hindering male involvement in the PMTCT program in the study area. The finding of this study was comparable to the findings of studies conducted in Lemo District Ethiopia, Uganda and Tanzania [19, 22, 25]. In this study, those who had low cultural **barriers** were 2.20 times more likely involved in PMTCT than those who had high cultural **barriers**. The possible explanation might be those males who had low cultural **barriers** can accompany their partners during all maternal and child health services, communicate with their wives freely about the service obtained from the PMTCT program. Tanzania, a social and religious norm prohibited males from attending female health services and the widespread attitude that female reproductive health is not male responsibility and found to inhibit male involvement in PMTCT [31]. And also males who had cultural barriers could perceive themselves at risk or have an experience that increases the risk of acquisition of HIV they become more discouraged to join hands with a partner in a PMTCT endeavor.

Respondents who had low health system **barriers** were 2.40 times more likely involved in PMTCT services when compared to their counterparts. The findings of the study are consistent with the study findings from Uganda, Lemo District and, Debremarkos Ethiopia [19, 22, 30]. The possible justification for this could be those males who had low health system **barriers** will have the more a man comes in contact with the health facility with his partner that helps male partners to have information about PMTCT.

### Study limitations

Due to the cross-sectional nature of this study, establishing a true cause and effect relationship between adherence status and associated factors would be impossible. This study might also suffer from recall bias.

## Conclusion

The level of male involvement in PMTCT programs in Enebsiesarmider District, Northwest Ethiopia was low (26.1%) in 2018. Having secondary education and above, good knowledge of PMTCT/ANC, a high level of cultural and health system barriers were significantly associated with male involvement in PMCT. Therefore, the regional health bureau, district health office in collaboration with local health care providers should work on coordinated and targeted IEC and BCC programs to convince male partners to utilize PMTCT service and develop a strategy for community mobilization. Furthermore, enhancing male involvement through creating a husband’s knowledge regarding the merit of prevention of mother to child transmission through the provision of adequate information for all male partners at the ANC clinic is recommended.

## Data Availability

The full data set and other materials about this study can be obtained from the corresponding author on reasonable request.

## Abbreviations

ANC: Antenatal Care
HEW: Health Extension Worker
PMTCT: Prevention of Mother to Child Transmission
SPSS: Statistical package for social science
WHO: World Health Organization
